# Integrins and Their Extracellular Matrix Ligands in Lymphangiogenesis and Lymph Node Metastasis

**DOI:** 10.1155/2012/853703

**Published:** 2012-02-12

**Authors:** Jie Chen, J. Steven Alexander, A. Wayne Orr

**Affiliations:** ^1^Department of Pathology, LSU Health Sciences Center, Shreveport, 1501 Kings Highway, Shreveport, LA 71130, USA; ^2^Department of Physiology, LSU Health Sciences Center, Shreveport, 1501 Kings Highway, Shreveport, LA 71130, USA

## Abstract

In the 1970s, the late Judah Folkman postulated that tumors grow proportionately to their blood supply and that tumor angiogenesis removed this limitation promoting growth and metastasis. Work over the past 40 years, varying from molecular examination to clinical trials, verified this hypothesis and identified a host of therapeutic targets to limit tumor angiogenesis, including the integrin family of extracellular matrix receptors. However, the propensity for some tumors to spread through lymphatics suggests that lymphangiogenesis plays a similarly important role. Lymphangiogenesis inhibitors reduce lymph node metastasis, the leading indicator of poor prognosis, whereas inducing lymphangiogenesis promotes lymph node metastasis even in cancers not prone to lymphatic dissemination. Recent works highlight a role for integrins in lymphangiogenesis and suggest that integrin inhibitors may serve as therapeutic targets to limit lymphangiogenesis and lymph node metastasis. This review discusses the current literature on integrin-matrix interactions in lymphatic vessel development and lymphangiogenesis and highlights our current knowledge on how specific integrins regulate tumor lymphangiogenesis.

## 1. Introduction to the Lymphatic Circulation

Blood vessels supply tissues with nutrients and oxygen, remove waste products, and provide a mechanism for leukocyte homing. Capillary exchange is vital for this process. As blood pressure causes fluid extravasation in the arterial side of the capillary bed, colloid osmotic pressure drives resorption of the fluid on the venous side. However, ~10% of this fluid is retained in the tissue accumulating as interstitial fluid [1]. During inflammation and tumorigenesis, this accumulation of interstitial fluid is augmented due to enhanced permeability of the capillaries resulting in tissue edema [2].

The lymphatic system regulates the transfer of interstitial fluid and cells from the tissue back into the circulation [1]. Nearly all vascularized tissues contain lymphatics with the exception of the bone marrow, retina, and brain [3, 4]. Disrupting lymphatic vessel function, due to either primary (genetic) or secondary (infectious, vessel damage) mechanisms, causes chronic tissue edema. In addition to fluid transport, the lymphatic circulation plays a vital role in the inflammatory response. Antigen-presenting cells such as macrophages and dendritic cells encounter antigen at sites of local tissue inflammation. Endothelial cells in the lymphatic capillaries produce CCL21 [5], a chemokine that stimulates antigen-presenting cells to migrate into the lymphatic capillaries [6]. Targeting to the lymphatics and subsequently to the lymph nodes allows antigen-presenting cells to interact with T cells and B cells, a key step in adaptive immunity [7].

**Figure 1:**
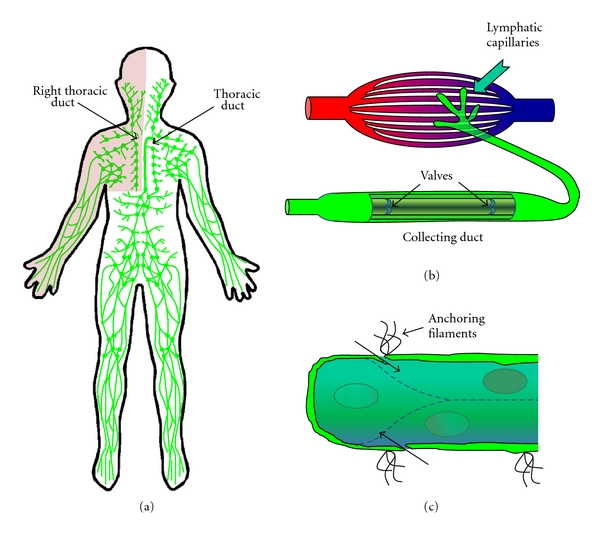
Lymphatic system structure. (a) The lymphatic system is separated into two distinct sets of tubules. Lymphatic vessels drain various areas of the body passing the material through a series of lymph nodes before returning the material to the venous circulation through the thoracic ducts. (b) Lymphatic capillaries drain interstitial fluid that accumulates during capillary exchange. The protein and cell-rich fluid termed lymph is then transported into vein-like valved collecting tubules. (c) Anchoring filaments couple lymphatic capillary endothelial cells to the surrounding matrix. Forces applied through these anchoring filaments enhance lymphatic permeability to promote tissue drainage.

While similar in composition, lymphatic vessels and blood vessels show some striking differences. Unlike the continuous vascular circulation, the lymphatic vessels are divided into two distinct lymphatic trees (Figure 1(a)). Lymphatic vessels in the head, thorax, and right arm drain into the right lymphatic trunk and empty into the right subclavian vein. The lymphatics in the lower limbs, abdomen, and left arm drain into the thoracic duct and empty into the left subclavian vein [8]. Lymphatic capillaries are closed ended tubes that lack a normal subendothelial basement membrane and show no smooth muscle cell or pericyte coverage [9, 10]. The material collected by these lymphatic capillaries, termed lymph, is driven into the collecting lymphatic vessels by interstitial pressure. Collecting vessels resemble venous vessels in that both have a subendothelial basement membrane, smooth muscle cells, and bileaflet valves which prevent fluid backflow (Figure 1(b)). Intrinsic contractility of lymphatic smooth muscle and skeletal muscle contractions propel the lymph forward where it is eventually returned to the venous circulation via the thoracic ducts [10]. Cell-cell junctions of lymphatic endothelial cells (LECs) are discontinuous and “button-like” allowing for a high degree of permeability in these vessels [11, 12]. Elevated interstitial pressure creates tension on LEC anchoring filaments enhancing LEC permeability and interstitial fluid uptake (Figure 1(c)). 

## 2. Lymphangiogenesis in Cancer

Metastatic tumor spread is responsible for more than 90% of cancer mortality [32] and tumor access to blood and lymphatic vessels drives systemic metastasis. For multiple types of cancer, including melanoma and carcinoma of the breast, cervix, colon, and prostate, lymph node metastasis represents the first step in tumor dissemination [10, 33, 34]. For this reason, the presence of lymph node metastasis is a key determinant in tumor staging and the leading indicator of poor prognosis [35, 36]. Lymphatic vessel density (LVD), the product of both preexisting lymphatic vessels and new vessels arising from lymphangiogenesis, correlates with lymph node metastasis in a number of cancer models [37]. As such, cancers arising in regions possessing an already high LVD (e.g., tonsillar, tongue, head, and neck cancer) may not require lymphangiogenesis for subsequent lymph node metastasis [38]. In addition to vessel density, the location of the lymphatic vessels may be critical as intratumoral vessels have been reported as non-functional based on high intratumoral interstitial fluid pressures which collapse lymphatics [39]. These results suggest peritumoral lymphatics may serve as the primary site of lymphatic entry for metastatic cells.

### 2.1. VEGF-C and Tumor Lymphangiogenesis

**Figure 2:**
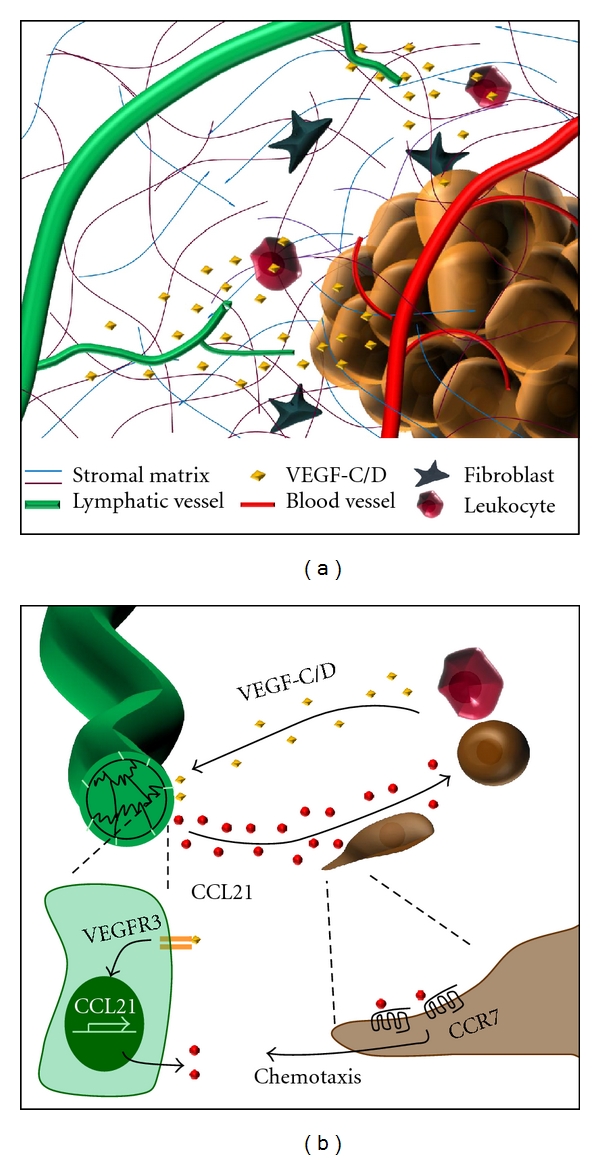
Local paracrine signaling controls lymphangiogenesis and lymph node metastasis. (a) Release of growth factors such as VEGF-C and VEGF-D by tumor and stromal cells promotes lymphatic endothelial cell sprouting, invasion, and capillary tube formation. (b) VEGF-C stimulates lymphatic endothelial cells to produce the chemokine CCL21. Expression of the CCL21 receptor on leukocytes and some tumor cells stimulates chemotaxis toward the lymphatic vessel promoting lymphatic dissemination.

Multiple growth factors modulate lymphangiogenesis, including hepatocyte growth factor (HGF), platelet-derived growth factor (PDGF), fibroblast growth factor (FGF), angiopoietin-1, endothelin-1, and members of the vascular endothelial cell growth factor (VEGF) family [40–48]. Several VEGF isoforms mediate tumor angiogenesis and VEGF/VEGF-receptor interactions have been targeted to modulate angiogenic responses [49]; the inhibitory anti-VEGF antibody *Avastin* was the first angiogenesis inhibitor to enter the market in 2004. The VEGF-A/VEGF-R2 interaction drives blood vessel angiogenesis, whereas lymphatic endothelial cells also express VEGF-R3 which shows higher affinity for VEGF-D and fully processed VEGF-C isoforms [41, 50]. Overexpression of VEGF-C or VEGF-D in mouse xenograft models enhances both lymphangiogenesis and lymph node metastasis [10, 51], and VEGF-C expression in human cancer correlates with enhanced lymphangiogenesis, lymph node metastasis, and poor prognosis (Figure 2(a)). Induction of skin carcinogenesis in transgenic mice overexpressing VEGF-C did not affect primary tumor size, but enhanced tumor metastasis to lymph nodes and the lung [52, 53]. Interestingly, VEGF-C overexpression enhanced lymph node metastasis even in xenografts from tumors that do not typically metastasize to lymph nodes [33, 51]. Perhaps most importantly, inhibitors of VEGF-C/VEGF-R3 signaling, including siRNA and soluble VEGF-R3, reduce lymphangiogenesis, lymph node metastasis, and enhance survival in mouse tumor models [54–56].

### 2.2. Lymphangiogenesis Inhibitors

Angiogenesis regulation involves the balance of proangiogenic and antiangiogenic factors. While many stimuli are known to activate lymphangiogenesis, less data exists describing the presence of endogenous lymphangiogenesis inhibitors (as has been described for angiogenesis). Mice deficient for the extracellular matrix protein thrombospondin-1 (TSP1), the first described endogenous inhibitor of angiogenesis [57], show exacerbated corneal lymphangiogenesis suggesting TSP1 may similarly inhibit lymphangiogenesis [58]. However, TSP1 overexpression does not show a similar antilymphangiogenic effect in skin carcinogenesis models presumably due to the absence of TSP1's antiangiogenic CD36 receptor in lymphatic endothelial cells [59]. Despite this, TSP1 may exert antilymphangiogenic activity indirectly by altering the levels of other lymphangiogenesis effectors. Consistent with a mostly indirect effect, the TSP1-activated growth factor TGF*β* actively suppresses lymphangiogenesis [60, 61], and TSP1-mediated CD36 ligation on corneal macrophages suppressed VEGF-C and VEGF-D expression [58]. Vasohibin and the collagen XVIII fragments endostatin and neostatin 7 reduce both angiogenesis and lymphangiogenesis [62–64], suggesting that these inhibitors target pathways common to both angiogenesis and lymphangiogenesis. Interestingly, a splice variant of VEGF-R2 encoding for a soluble form of the receptor did not affect tumor angiogenesis but blocked lymphangiogenesis presumably due to the ability of soluble VEGF-R2 to bind VEGF-C [65].

### 2.3. Cancer Cell Chemotaxis toward Lymphatic Chemokines

Cancer cells often enter lymphatics at the level of the lymphatic capillaries. This process is aided by the LECs themselves, which secrete chemokines such as CCL21 that induce chemotaxis in antigen-presenting cells and some cancer cells [5]. VEGF-C expressed by tumor cells and monocytes in the tumor stroma stimulates LEC production of CCL21, and CCL21 in turn activates its receptor CCR7 in cancer cells (Figure 2(b)) [66]. Xenografts of CCR7 expressing melanoma cells were found to grow towards regions of implanted LECs. Interestingly, only metastatic malignant melanoma cells express CCR7, while their nonmalignant counterparts do not [67, 68]. Similarly, breast cancer cells showing lymph node metastasis also show enhanced CCR7 expression [69], and breast cancer cell xenografts showed lymph node metastasis when CCR7 was expressed [70].

In addition to the CCL21/CCR7 axis, lymphatic endothelial cells also express SDF-1 which promotes metastasis to lymph nodes in several cancer cells that express the SDF-1 receptor CXCR4 (For a full review see [71]). PDGF-D overexpression stimulated CXCR4 expression in breast carcinoma xenografts and promoted lymph node metastasis [72]. Furthermore, a polymorphism in SDF-1*α* (G801A) which results in elevated SDF-1 expression was associated with lymph node metastasis and shorter survival time in patients with colorectal cancer [73]. 

## 3. The Integrin Family of Receptors and Their Extracellular Matrix Ligands

The extracellular matrix (ECM) is a highly organized complex of collagens, proteoglycans, glycoproteins, and growth factors capable of creating varying degrees of tissue tensile strength, from mucosal linings to bones. Laminins and collagen IV form a thin sheet-like matrix termed basement membrane that separates epithelial and endothelial cells from underlying connective tissue [74]. Fibrillar collagens (e.g., collagen I, collagen III) make up the bulk of the body's connective tissue and play a major role in regulating tissue tensile strength due to their capacity to be cross-linked into fibers. Although not normally involved in maintaining tissue structure, provisional and matricellular matrix proteins are rapidly deposited during tissue remodeling responses coordinating cell migration and proliferation to heal injured tissue. Provisional matrix proteins (e.g., fibronectin, fibrinogen, vitronectin) present in the bloodstream leak into wounded areas and provide an adhesive scaffold for the recruitment of cells [75, 76]. Matricellular matrix proteins (e.g., thrombospondin, tenascin-C, SPARC, osteopontin) generally play a minimal role in tissue structure but instead regulate the cell's interaction with structural matrix proteins and modulate cell function [77, 78]. Interactions with ECM proteins affect nearly every aspect of cellular physiology, from cell proliferation and migration, to gene expression and differentiation [79]. Specific cell-matrix interactions are critical for the survival of many cell types, and loss of this adhesion dependence is a classic hallmark of neoplastic change [80]. Furthermore, ECM proteins are secreted and organized by the cells in the local environment, suggesting that cells and their matrices exist in a state of “dynamic reciprocity” as each one serves to regulate the other [81].

### 3.1. Matrix Composition in the Tumor Stroma

During tumorigenesis, the expanding tumor stimulates the production of local supportive tissue termed the tumor “stroma” which is composed of proliferating fibroblasts, leukocytes, blood and lymphatic vessels, and ECM proteins. Mounting evidence suggests that the local tumor microenvironment plays a critical role in cancer progression from a collection of transformed cells to a clinically relevant disease [82]. During stromal matrix formation, cancer cells and stromal fibroblasts show enhanced deposition of fibrillar collagens (e.g., collagen I and III), provisional matrix proteins (e.g., fibronectin), and matricellular proteins (e.g., tenascin-C, osteopontin) [83, 84]. While the stromal matrix plays established roles in angiogenesis and tumor metastasis [85], mounting evidence suggests that the stromal matrix regulates tumor lymphangiogenesis as well.

### 3.2. The Integrin Family of Matrix Receptors

**Figure 3:**
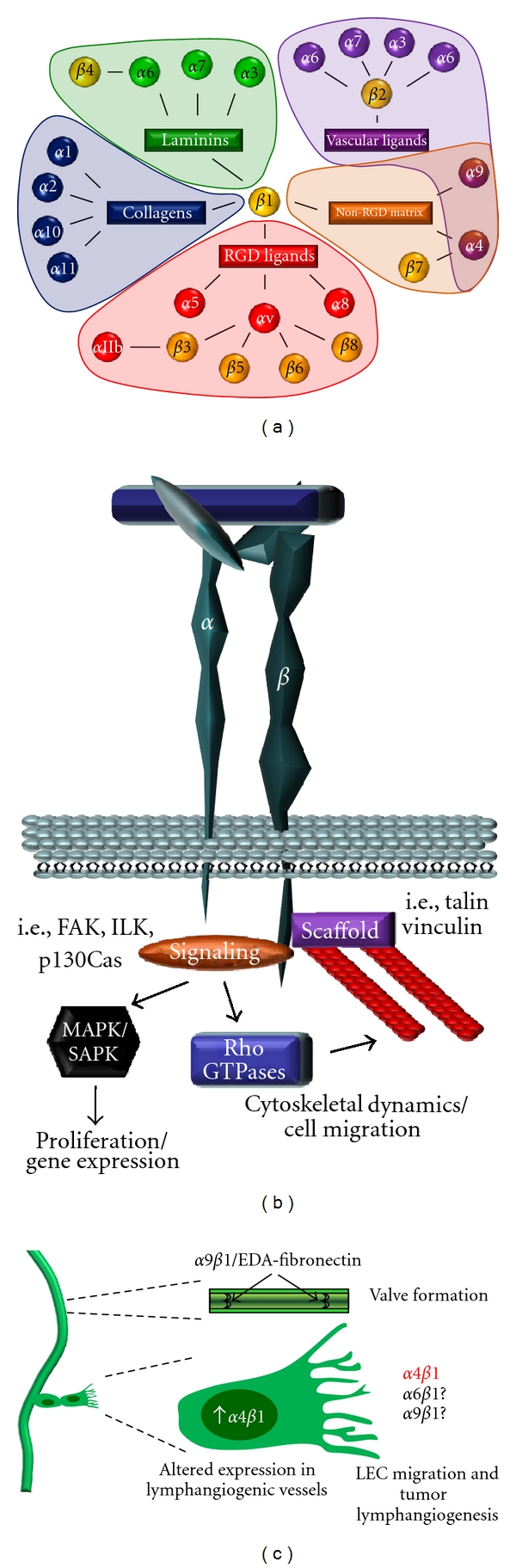
The integrin family of matrix receptors in lymphangiogenesis. (a) Integrin subunits divided by their binding partners (connecting lines) and ligand-binding preferences (shaded areas). (b) Structure of integrin adhesions. Integrins link the extracellular matrix to the intracellular actin cytoskeleton through structural adaptor proteins. Recruitment of signaling proteins activates pathways that regulate gene expression (e.g., MAP kinases) and cytoskeletal reorganization (Rho GTPases). (c) Expression of the integrin *α*9*β*1 and its EDA-fibronectin ligand are required for proper lymphatic valve development. While *α*6*β*1 and *α*9*β*1 are implicated in tumor angiogenesis, only *α*4*β*1 has been shown to be upregulated in lymphangiogenic vessels, to mediate LEC migration and tube formation in culture, and to be required for tumor-associated lymphangiogenesis.

Interactions between ECM components and the integrin family of matrix receptors serves to anchor cells to the underlying matrices, mechanically couple the actin cytoskeleton to the external environment, and activate a broad spectrum of integrin-specific signaling pathways. The integrin family exists as heterodimers of unique *α* and *β* subunits; mammals express 18 *α* and 8 *β* subunits forming 24 distinct *αβ* integrin dimers (Figure 3(a)). Integrin expression patterns depend on the specific cell type and vary with environmental context [86, 87]. Leukocyte homing responses typically involve a separate subset of integrins (*α*L*β*2, *α*M*β*2, *α*X*β*2, *α*D*β*2) that interact with counter-receptors on the endothelial cell surface such as ICAM-1 and VCAM-1. Distinct collagen-binding integrins (*α*1*β*1, *α*2*β*1, *α*10*β*1, *α*11*β*1) and laminin-binding integrins (*α*3*β*1, *α*6*β*1, *α*6*β*4) serve to anchor cells to the basement membrane and interstitial matrices. Provisional matrix proteins often contain RGD sequences that mediate interactions with *α*5*β*1, *α*v*β*3, and *α*v*β*5 among others. While the affinity for an RGD sequence is a common theme for provisional matrix binding integrins, there are exceptions to this trend such as the related integrins *α*4*β*1 and *α*9*β*1. The *α*4*β*1/*α*9*β*1 integrin subfamily interacts with both components of the provisional matrix (e.g., fibronectin CS-1 and EDA domains, tenascin-C, osteopontin) and vascular ligands involved in leukocyte homing (VCAM-1).

### 3.3. Integrin Signaling

As cells contact the ECM, the integrin extracellular domains bind to their ligands anchoring the cell to the matrix and altering the integrin cytoplasmic domain structure (Figure 3(b)). Structural proteins such as talin and vinculin serve as bridges between the integrin cytoplasmic tail and the actin cytoskeleton [88]. Although the integrin cytoplasmic domain lacks intrinsic enzymatic activity, the structural alteration assumed following integrin ligation stimulates interactions with intracellular signaling proteins [79, 89]. Integrin cytoplasmic domains differ considerably between individual integrin subunits allowing for integrin-specific signaling responses, although some motifs are common [79, 90]. Ligated integrins recruit several nonreceptor tyrosine kinases, including focal adhesion kinase (FAK), integrin-linked kinase (ILK), and Src-family kinases, among others [79, 90]. Integrin adhesion regulates signaling through the Rho family of small GTPases including Rac, cdc42, and Rho resulting in cytoskeletal changes associated with cell migration [91]. In addition, integrin signaling activates multiple signaling pathways that affect gene expression patterns, such as the MAP kinases (ERK, JNK, and p38) and the transcription factors c-fos, c-jun, and NF-*κ*B [79].

## 4. Cell Matrix Interactions in Lymphangiogenesis

### 4.1. Extracellular Matrix of the Tumor Stroma and Lymphangiogenesis

Although abundant in the tumor stroma, collagen's role in tumor lymphangiogenesis remains unclear. A recently identified protein termed collagen and calcium-binding EGF domain-1 (CCBE1) is essential for developmental lymphangiogenesis in both zebrafish and mouse models [92, 93]. Although little is currently known about CCBE1, it appears to bind to collagen and vitronectin in the extracellular matrix, and a lack of CCBE1 expression prevents the budding of new lymphatic endothelial cells from the cardinal vein [93]. However, collagen's role in mediating the lymphangiogenic effects of CCBE1 has yet to be explored.

While multiple provisional matrix and matricellular proteins are implicated in tumor lymphangiogenesis, the most convincing data for matrix-dependent lymphangiogenesis involves the provisional matrix protein fibronectin. The fibronectin gene can undergo alternative splicing to include three additional sites: the connecting segment-1 (CS-1), extra domain A (EDA), and extra domain B (EDB) [94]. Fibronectin in the tumor stroma often contains the CS-1 and EDA domains [95, 96]. Blocking antibodies against the EDA site reduce LEC expression of Prox1 and F-actin, key regulators of lymphangiogenesis [95]. In contrast to the CS-1 and EDA domains, the EDB site in fibronectin has not yet been implicated in the lymphangiogenic process. In addition to fibronectin, tenascin-C and osteopontin expression in the tumor stroma is associated with enhanced lymph node metastasis [97, 98], and LECs upregulate tenascin-C expression during lymphangiogenesis [99]. Taken together, these data show that the ECM composition in the tumor stroma is a critical regulator of both lymphangiogenesis and lymph node metastasis.

### 4.2. Interactions with Anchoring Filaments in Lymphatic Development

Lymphatic capillary endothelial cells share some similarities with vascular endothelial cells with the exception of the absence of a continuous basement membrane and surrounding pericytes. Instead of adhering to the basement membrane, capillary LECs are attached to anchoring filaments composed of fibrillin and emilin-1 which anchor the lymphatic capillaries to the surrounding collagen filaments in the interstitial matrix (Figure 1(c)) [100, 101]. This allows for coupling of interstitial fluid pressure changes to the LEC cytoskeleton such that increased interstitial pressure increases permeability of the lymphatic capillaries to enhance drainage of interstitial fluid. Lymphatic vessels express multiple integrins including *α*1*β*1, *α*2*β*1, *α*3*β*1, *α*4*β*1, *α*5*β*1, *α*6*β*1, *α*v*β*3, and *α*9*β*1 (Table 1) [13, 20, 30, 42, 102–104], and LECs appear to utilize multiple integrins to interact with these anchoring filaments. Fibrillin stimulates LEC adhesion through the RGD-binding integrins *α*5*β*1 and *α*v*β*3 [105]. However, the importance of *α*5*β*1 and *α*v*β*3 to lymphatic endothelium is questionable, since mice deficient for both *α*5 and *α*v integrin subunits in endothelial cells show no apparent developmental defects in lymphangiogenesis or lymphatic function [21]. Mutations in fibrillin genes are associated with Marfan's syndrome, and fibrillin knockout mice recapitulate this phenotype [106]. However, no lymphatic phenotype has been described to date associated with either Marfan's syndrome or fibrillin knockout mice. In contrast, mice deficient for the anchoring filament protein emilin-1 show reduced numbers of anchoring filaments [107], as well as hyperplastic and dysfunctional lymphatic vessels. The integrin *α*4*β*1, classically associated with leukocyte homing to regions of inflammation, is the only known receptor for emilin-1 to date [108]. However, again, no defects in developmental lymphangiogenesis were described in mice lacking endothelial *α*4 integrins or expressing a dominant negative form of *α*4 (Y991A) deficient in talin and paxillin binding [29].

### 4.3. Integrin *α*9*β*1 in Lymphatic Development

In contrast to *α*4*β*1, *α*5*β*1, and *α*v*β*3 integrins, the expression of *α*9*β*1 integrins in LECs is crucial to the process of developmental lymphangiogenesis. The lymphatic network arises by the initial segregation of a discrete endothelial cell population from the cardinal vein [4]. This early transition from venous endothelium to lymphatic endothelium is driven by the homeobox transcription factor Prox1. Prox1 is required for sprouting and migration of LECs toward lymphatic growth factors, for example, VEGF-C and -D [109]. In mouse embryos, Prox1 drives the expression of VEGFR3 and *α*9 integrin in the newly forming LECs [110]. Mice deficient for *α*9 integrin die postnatally due to lung chylothorax, an accumulation of lymph in the pleural cavity [22, 23]. Interestingly, a missense mutation in the human *α*9 integrin gene is associated with congenital chylothorax in human fetuses [24]. The extracellular matrix ligand for *α*9*β*1 during lymphatic development remains unclear, since multiple matrix proteins can interact with *α*9*β*1, including tenascin-C, the EDA domain of fibronectin, and osteopontin (Figure 3(c)). However, fibronectin appears to be the dominant ligand for *α*9*β*1-dependent lymphatic valve formation. EDA-positive fibronectin deposition occurs early during lymphatic valve formation in an *α*9*β*1 integrin-dependent manner [22]. Mice deficient in either *α*9*β*1 knockout or EDA-positive fibronectin show similar defects in lymphatic valve formation [22, 111]. Taken together, these data illustrate a major role for *α*9*β*1 integrin and its matrix ligand EDA-positive fibronectin in lymphatic development.

### 4.4. Integrins in Inflammatory Lymphangiogenesis

Multiple integrins have been implicated in pathological lymphangiogenesis. However, these studies are often limited to a single model system providing little insight into their relevance to tumor lymphangiogenesis. For example, VEGF-A stimulates expression of the collagen-binding integrins *α*1*β*1 and *α*2*β*1 in LECs [13], and blockade of these integrins using antibodies prevents lymphangiogenesis in both wound healing and corneal inflammation models [13, 14]. However, the role of these integrins in tumor lymphangiogenesis has not yet been addressed. The provisional matrix binding integrins *α*5*β*1, *α*v*β*3, and *α*v*β*5 mediate tumor angiogenesis, and inhibitors to these integrins are currently being tested in clinical trials [17–19]. However, the role these integrins play in tumor lymphangiogenesis is less clear. The fibronectin-binding integrin *α*5*β*1 shows enhanced expression in lymphatic sprouts [15], and fibronectin can induce LEC proliferation in culture [103]. Blocking the *α*5*β*1 integrin with small molecule inhibitors JSM6427 and JSM8757 significantly blunts lymphangiogenesis in corneal inflammation and airway inflammation models [15, 16]. Despite these findings, *α*5*β*1 does not appear to be involved in tumor lymphangiogenesis [20]. Furthermore, *α*v*β*3 and *α*v*β*5 show only minimal expression in LECs and do not appear to be involved in lymphangiogenic responses [20].

### 4.5. *α*9*β*1 and *α*4*β*1 Integrins Mediate Tumor Lymphangiogenesis

Because *α*9*β*1 integrin has an established role in developmental lymphangiogenesis, it likely also participates in tumor angiogenesis as well, and several lines of evidence support this. VEGF-C and VEGF-D are key mediators of tumor lymphangiogenesis and *α*9*β*1 binds to the EYP sequence in VEGF-A, C, and D to promote endothelial and tumor cell migration [25, 26]. Consistent with this, *α*9*β*1 blocking antibodies were shown to suppress VEGF-C-induced chemotaxis in LECs [26]. The angiogenic suppressor endostatin also reduces lymphangiogenesis in colorectal and skin squamous cell carcinomas [27, 28] and inhibits lymph node metastasis [27]. Interestingly, endostatin was recently shown to block interactions between *α*9*β*1 and the EDA domain of fibronectin [28]. However, endostatin can also inhibit fibronectin interactions with *α*5*β*1 [112], suggesting that endostatin's effects may not be solely mediated by *α*9*β*1. As such, no studies to date have definitively proven that *α*9*β*1 plays a functional role in tumor lymphangiogenesis *in vivo*.

While quiescent lymphatic endothelial cells weakly express *α*4*β*1, lymphatic vessels associated with variety of human and murine tumors show enhanced *α*4*β*1 expression. The lymphangiogenic/angiogenic growth factors VEGF-A, VEGF-C, and bFGF all induce *α*4*β*1 expression in lymphangiogenic vessels, whereas proliferating LECs *in vitro* show high levels of *α*4*β*1 expression [20, 29]. Both *α*4*β*1-blocking antibodies and recombinant soluble VCAM-1 suppress lymphangiogenesis induced in VEGF-A or VEGF-C infused matrigel plugs and lead to elevated LEC apoptosis [29]. Blocking antibodies to *α*4*β*1, but not *α*5*β*1, *α*v*β*3, or *α*v*β*5, suppressed VEGF-C-induced LEC migration, matrigel invasion, and tube formation (Figure 3(c)). Endothelial-specific *α*4 integrin knockout mice showed significantly reduced lymphangiogenesis in VEGF-C infused matrigel plugs. Mutating the Y991A in the *α*4-cytoplasmic tail disrupts paxillin binding and inhibits leukocyte homing [113]. LECs isolated from *α*4 Y991A transgenic mice show reduced migration to VEGF-C, and VEGF-C-induced lymphangiogenesis was reduced in *α*4 Y991A transgenic mice [29]. Treatment with *α*4*β*1 blocking antibodies reduced lymphangiogenesis and lymph node metastasis in implanted Lewis lung carcinoma and B16 melanoma tumors. However, bone marrow transplant experiments using wild-type and *α*4 Y991A knock-in mice suggested that *α*4*β*1 inhibition in either recipient or donor cells reduces lymphangiogenesis. Therefore, *α*4*β*1 inhibitors may interfere with lymphangiogenesis by both inhibiting LEC migration and preventing homing of proangiogenic leukocytes [29].

Recent evidence suggests the laminin-binding integrin *α*6*β*1 may play a role in tumor lymphangiogenesis as well. The netrin family of axonal guidance molecules are secreted laminin-like proteins implicated in angiogenesis and tumor metastasis [114]. Lymphatic vessels associated with breast tumors express netrin-4, and LECs show enhanced proliferation, migration, and tube formation in response to netrin-4 (Figure 3(c)) [31]. Overexpression of netrin-4 increases LVD in mouse skin, and breast cancer xenografts overexpressing netrin-4 show enhanced LVD and metastasis [31]. Netrin-4 binding to *α*6*β*1 cooperatively enhances binding between *α*6*β*1 and laminin, suggesting netrin-4 directly modulates *α*6*β*1 activation [30]. Inhibition of *α*6*β*1 blocks LEC migration on netrin-4, and *α*6*β*1 colocalizes with netrin-4 in lymphatic vessels during embryogenesis, in adult intestine, and in breast tumor xenografts [30]. However, a direct causal role of *α*6*β*1 signaling in netrin-4-associated lymphangiogenesis has yet to be determined.

## 5. Clinical Perspective: Targeting Lymphangiogenesis with Integrin Inhibitors

Several integrin inhibitors have made their way into the clinic, and a new wave of integrin inhibitors are advancing through clinical trials (Table 1). Current integrin inhibitors fall into three categories: therapeutic antibodies, ligand-mimetic peptides, and small molecule antagonists [17–19]. To date, the only FDA-approved integrin inhibitors have targeted the integrin *α*4 (natalizumab) and platelet integrin *α*IIb*β*3 (abciximab, eptifibatide, tirofiban) [18]. Most of the inhibitors currently in clinical trials target the RGD-binding integrins *α*5*β*1, *α*v*β*3, *α*v*β*5, and *α*IIb*β*3 which do not appear to significantly modulate lymphatic function [21]. The *α*v*β*3/*α*v*β*5 inhibitor cilengitide is the agent closest to approval, with Phase III clinical trials for glioblastoma ongoing. There are no current clinical trials specifically testing the efficacy of integrin inhibitors in lymphangiogenesis and lymph node metastasis. Still, lymphangiogenesis itself is only specifically targeted by a handful of trials, and these tend to focus on the role of growth factor signaling in lymphangiogenesis. While *α*9 is closely associated with lymphatic development and LEC migration, the best data for integrin involvement in lymphangiogenesis involves the integrin *α*4*β*1 [20, 29], and an inhibitor of the *α*4-integrin natalizumab (Tysabri) has been approved for the treatment of chronic inflammatory diseases such as multiple sclerosis and Crohn's disease since 2004 [115]. While this approval was quickly recalled following multiple cases of progressive multifocal leukoencephalopathy in a subset of patients, the benefits of natalizumab for multiple sclerosis patients were found to outweigh the potential risks and the drug was again approved for use in the USA in 2006 [116].

Targeting the *α*4*β*1 and *α*9*β*1 integrins for therapeutic reduction in lymphangiogenesis would be predicted to reduce inflammation in the tumor, as both *α*4*β*1 and *α*9*β*1 are known to regulate leukocyte homing responses. However, the benefits of this potential off target effect are unclear since inflammation plays a complex role in tumor formation, progression, and metastasis [117, 118]. The tumor stroma contains both tumor-associated macrophages and lymphocytes. Tumor-associated macrophages, generally alternatively activated M2 macrophages, produce a variety of cytokines and growth factors that promote tumor growth and reduce apoptosis [119]. Additionally, tumor-associated macrophages promote tumor metastasis by enhancing ECM degradation in the tumor stroma, promoting angiogenesis, and stimulating endothelial adhesion molecule expression to allow extravasation [117, 120]. As such, the presence of a high number of tumor-associated macrophages is associated with poor prognosis in multiple cancers [121]. Therefore, integrin inhibitors that could restrict both inflammation and lymphangiogenesis/lymph node metastasis may prove beneficial. Consistent with this, integrin *α*4*β*1 antagonists suppress macrophage colonization of tumors and subsequent tumor angiogenesis [122]. Alternatively, adaptive immunity plays a well-accepted role in immunosurveillance that is thought to limit the growth of some tumors, including many of those prone to lymph node metastasis such as colon cancer and melanoma [118, 123]. In this case, inhibiting *α*4*β*1 and *α*9*β*1 might be expected to propagate tumor formation. Therefore, differences in tumor type and stage, immunogenicity, and tendency for lymph node metastasis are likely to influence when and how *α*4*β*1 and *α*9*β*1 integrin inhibitors can be used in cancer therapy.

## Figures and Tables

**Table 1:** Integrins in lymphangiogenesis.

LEC integrins	Matrix ligands	Data implicating integrin in lymphangiogenesis	Current inhibitors FDA approved or in clinical trials
*α*1*β*1, *α*2*β*1	Collagens	Overexpressed in LEC treated with VEGF-A [13]; blocking antibodies reduce lymphangiogenesis in wound healing [13]; corneal inflammation models [14]	None

*α*5*β*1	Fibronectin	Expressed in sprouting LECs [15]; small molecule inhibitors reduce lymphangiogenesis in cornea [15]; lung inflammation models [16]	Volociximab PF-04605412 JSM6427 [17–19]

*α*v*β*3, *α*v*β*5	Fibronectin (RGD), Osteopontin, Vitronectin, Fibrinogen Fibrillin	Minimal expression in LECs [20]; no role in lymphangiogenesis described to date [20, 21]	Cilengitide, CNTO95 EMD525797 IMGN388 [17–19]

*α*9*β*1	Fibronectin (EDA), Osteopontin, Tenascin-C, VEGF-A/C/D	Knockout mice die postnatally due to defective lymphatic valve development (lung chylothorax) [22–24]; binds directly to VEGF-A/C/D and blocking antibodies inhibit LEC migration [25, 26]; endostatin (*α*5*β*1 and *α*9*β*1 inhibitor) blocks lymphangiogenesis in cancer models [27, 28]	None

*α*4*β*1	Fibronectin (CS1), Osteopontin, Emillin-1	Not required for developmental lymphangiogenesis [29]; expressed in tumor-associated lymphangiogenic vessels and in proliferating LECs [20, 29]; blocking antibodies prevent VEGF-C-induced LEC migration [20, 29]; knockout and dominant negatives block tumor lymphangiogenesis [29]	Natalizumab Vedolizumab ELND002 [17–19]

*α*6*β*1	Laminin, Netrin-4	Mediates LEC adhesion and migration to prolymphangiogenic factor Netrin-4 [30, 31]; colocalizes with netrin-4 in lymphangiogenic vessels associated with breast tumor xenografts [30]	None
